# Manipulation of diacylglycerol and ERK-mediated signaling differentially controls CD8^+^ T cell responses during chronic viral infection

**DOI:** 10.3389/fimmu.2022.1032113

**Published:** 2022-11-24

**Authors:** Shohei Harabuchi, Omar Khan, Hamid Bassiri, Taku Yoshida, Yohei Okada, Masaomi Takizawa, Osamu Ikeda, Akihiro Katada, Taku Kambayashi

**Affiliations:** ^1^ Department of Pathology and Laboratory Medicine, Perelman School of Medicine at the University of Pennsylvania, Philadelphia, PA, United States; ^2^ Department of Otolaryngology-Head and Neck surgery, Asahikawa Medical University, Asahikawa, Japan; ^3^ Department of Laboratory Medicine, University of California, San Francisco, San Francisco, CA, United States; ^4^ Division of Infectious Diseases, Department of Pediatrics, Perelman School of Medicine at the University of Pennsylvania, Philadelphia, PA, United States; ^5^ Immuno-Oncology, Astellas Pharma Inc., Tsukuba, Japan; ^6^ Research Program Management-Applied Research Management, Astellas Pharma Inc., Tokyo, Japan

**Keywords:** TCR T cell receptor, diacylglycerol kinase (DGK), ERK (extracellular signal-regulated kinase), chronic viral infection, T cell exhaustion

## Abstract

**Introduction:**

Activation of T cell receptor (TCR) signaling is critical for clonal expansion of CD8+ T cells. However, the effects of augmenting TCR signaling during chronic antigen exposure is less understood. Here, we investigated the role of diacylglycerol (DAG)-mediated signaling downstream of the TCR during chronic lymphocytic choriomeningitis virus clone 13 (LCMV CL13) infection by blocking DAG kinase zeta (DGKζ), a negative regulator of DAG.

**Methods:**

We examined the activation, survival, expansion, and phenotype of virus-specific T cell in the acute and chronic phases of LCMV CL13-infected in mice after DGKζ blockade or selective activation of ERK.

**Results:**

Upon LCMV CL13 infection, DGKζ deficiency promoted early short-lived effector cell (SLEC) differentiation of LCMV-specific CD8+ T cells, but this was followed by abrupt cell death. Short-term inhibition of DGKζ with ASP1570, a DGKζ-selective pharmacological inhibitor, augmented CD8+ T cell activation without causing cell death, which reduced virus titers both in the acute and chronic phases of LCMV CL13 infection. Unexpectedly, the selective enhancement of ERK, one key signaling pathway downstream of DAG, lowered viral titers and promoted expansion, survival, and a memory phenotype of LCMV-specific CD8+ T cells in the acute phase with fewer exhausted T cells in the chronic phase. The difference seen between DGKζ deficiency and selective ERK enhancement could be potentially explained by the activation of the AKT/mTOR pathway by DGKζ deficiency, since the mTOR inhibitor rapamycin rescued the abrupt cell death seen in virus-specific DGKζ KO CD8+ T cells.

**Discussion:**

Thus, while ERK is downstream of DAG signaling, the two pathways lead to distinct outcomes in the context of chronic CD8+ T cell activation, whereby DAG promotes SLEC differentiation and ERK promotes a memory phenotype.

## Introduction

CD8^+^ T cells are important in defense against viral infections and cancer. CD8^+^ T cells are exposed to persistent antigen and inflammatory signals during chronic infections and cancer. This continuous exposure induces the dampening of T cell function, termed T cell exhaustion (1–8). Exhausted CD8^+^ T cells express an altered transcriptional program that leads to a loss of robust effector function and to the persistent expression of multiple inhibitory receptors (2). CD8^+^ T cell exhaustion was initially reported more than two decades ago in mice infected with a chronic form of lymphocytic choriomeningitis virus (LCMV) (3, 4). Subsequently, CD8^+^ T cell exhaustion was demonstrated in other animal models and in humans with chronic viral (HIV, HBV, HCV), bacterial, and parasitic infections as well as in cancer (6).

LCMV infection has become a staple system for testing and discovering fundamental immunological concepts since its use in the 1930s (9), and the ability of an LCMV variant to establish chronic infection was critical for the discovery of T cell exhaustion. In contrast to the parental acute strain (LCMV Armstrong), the lysine to glutamine L1079 mutation in LCMV Clone 13 (LCMV CL13) isolate allows it to replicate more rapidly, increasing the levels of antigen, and inducing higher and longer lasting viremia (9, 10). The LCMV CL13-induced prolonged antigen persistence and inflammatory environment induced by LCMV CL13 elicits T cell exhaustion.

Although many of the receptors involved and the events that participate in the induction of T cell exhaustion are well-studied, how early T cell activation affects the outcome of T cell responses in chronic viral infection are less well understood. During the first week of viral infection, virus-specific T cells expand in secondary lymphoid organs through stimulation of their T cell receptor (TCR) by viral antigens presented by antigen presenting cells. One of the most important proximal signaling events that occurs downstream of TCR engagement is the activation of phospholipase Cγ1 (PLCγ1). PLCγ1 is an enzyme that hydrolyzes phosphatidylinositol 4,5-bisphosphate (PIP_2_) to generate two second messengers, inositol 1,4,5-trisphosphate (IP_3_) and diacylglycerol (DAG). DAG is essential for the activation of diverse downstream signaling cascades including the Ras-extracellular signal-related kinase (ERK)-activator protein (AP)-1 pathway, the protein kinase C (PKC)θ-IκB kinase (IKK)-nuclear factor (NF)-κB pathway, and the AK strain transforming (AKT)-tuberous sclerosis complex (TSC)1/2-mammalian target of rapamycin (mTOR) pathway (11, 12).

DAG signaling is negatively regulated by diacylglycerol kinases (DGK), which phosphorylate DAG, converting it into phosphatidic acid (PA) (12–14). DGKζ, one of the isoforms of DGK, serves as a critical negative regulator of DAG signaling and can modulate the strength of TCR signaling (15). Although DGKζ-deficient mice harbor normal numbers of thymocytes and splenocytes (16), DGKζ does affect T cell function. For example, overexpression of DGKζ inhibits TCR signaling by reducing the levels of active GTP-bound Ras and, consequently, diminishing ERK activation (17). Conversely, CD8^+^ T cells deficient in DGKζ display increased ERK activation and heightened cytotoxicity and cytokine production, leading to enhanced responsiveness towards cancer (18) and acute viral infection (16, 19).

Here, we tested how DAG signaling affects T cell responses during chronic infection with LCMV CL13. We find that enhanced DAG signaling leads to rapid expansion of LCMV-specific T cells, which is followed by enhanced Bim expression and complete collapse of this T cell pool at later stages of infection. Surprisingly, however, selective activation of ERK similarly leads to rapid expansion of LCMV-specific T cells without causing cell death, and results instead in T cells with a memory phenotype. Using ASP1570, a DGKζ-specific inhibitor, we find that short term inhibition of DGKζ in the acute and chronic phases of LCMV CL13 infection enhances activation of CD8^+^ T cells and decreases viral titers without causing cell death. Thus, our data demonstrate how different early signaling pathways differentially affect the fate of T cells during chronic infection and yields insight into new immunnotherapeutic targets and strategies.

## Materials and methods

### Mice

C57BL/6 (WT mice; CD45.1^−^CD45.2^+^CD90.1^−^CD90.2^+^), B6.SJL-PtprcaPepcb/BoyCrCrl (SJL mice; CD45.1^+^CD45.2^−^CD90.1^−^CD90.2^+^) mice, and B6.PL-Thy1a/CyJ (Thy1.1 mice; CD45.1^−^CD45.2^+^CD90.1^+^CD90.2^−^) were purchased from the Jackson laboratory or Charles River Laboratories. Generation of DGKζ KO mice were described previously (20, 21). Sevenmaker (ERK^SEM^) (CD45.1^−^CD45.2^+^CD90.1^−^CD90.2^+^) mice were provided by L. Samuelson from the National Institutes of Health and were originally developed by S. Hedrick from the University of California, San Diego (22). Wild type P14 mice (CD45.1^−^ CD45.2^+^CD90.1^−^CD90.2^+^) were provided by E.J. Wherry (1). SJL mice were crossed to P14 mice to generate WT-P14 (CD45.1^+^CD45.2^+^CD90.1^−^CD90.2^+^ or CD45.1^+^ CD45.2^−^CD90.1^−^CD90.2^+^). DGKζ KO mice and ERK^SEM^ mice were crossed to P14 mice to generate DGKζ KO-P14 and ERK^SEM^-P14 (CD45.1^−^CD45.2^+^CD90.1^−^CD90.2^+^). Unless otherwise specified, all mice were 7 to 12 weeks old at the time of use, were housed in pathogen-free conditions, and were treated in strict compliance with the Institutional Animal Care and Use Committee regulations at the University of Pennsylvania.

### Flow cytometry

For flow cytometric analyses, cells were stained with antibodies against cell surface antigens at 4°C for 30 min in phosphate-buffered saline (PBS). LIVE/DEAD Fixable near-IR Dead Cell Stain Kit was used to exclude nonviable cells. Intracellular cytokine staining was performed with the BD Cytofix/Cytoperm Kit according to the manufacturer’s protocol. Flow cytometry was performed with an LSR II or LSR Fortessa or FACS Canto flow cytometer (BD Biosciences). Data were analyzed using FlowJo software (TreeStar). All fluorochrome-conjugated anti- bodies are listed in **Table S1**. The gating strategy for tetramer^+^CD8^+^ T cells is shown in **Supplementary Figures 1A, B**.

### CD8^+^ T cell functional assays

For CD8^+^ T cell activation assays, freshly isolated splenocytes from LCMV CL13-infected mice were cultured together with anti-CD107a Ab and monensin for 5 h in tissue culture plates. During 5 h in culture, cells were restimulated with GP33 peptide (200 ng/ml; GP_33-41_), peptide pool (mixed for 200 ug/ml each; GP_33-41_, GPC_92-101_, GPC_118-125_, GPC_221-228_, GPC_276-286_, L_156-163_, L3_38-346_, L_349-357_, L_455-463_, L_775-782_, L_1428-1435_, L_2062-2069_, NP_165-175_, NP_205-212_, NP_238-248_, NP_396-404_), or PMA (100 ng/ml; Sigma-Aldrich) and ionomycin (1 mg/ml; Sigma-Aldrich). Peptides were provided E.J. Wherry (23). After 5 h in culture, CD8^+^ T cells were analyzed for anti-CD107a Ab staining and intracellular IFNγ by flow cytometry.

### Adoptive transfer of P14 T cells

TCR transgenic GP33-specific cells (P14) were isolated from the peripheral blood or splenocytes of donor mice using gradient centrifugation with Histopaque-1083 (Sigma-Aldrich) as previously described (1). For experiments using LCMV infection, CD45.1^+^ WT-P14 T cells were mixed 1:1 with CD45.1^−^ P14 T cells of the desired genotype (DGKζ KO -P14 and ERK^SEM^-P14) and a total of 0.5-1.0 × 10^3^ T cells were adoptively transferred by intravenous injection into 7-10-week-old recipient (Thy1.1) mice 1 day before infection as previously described (1). This enabled us to distinguish both donor populations from the host T cells (CD90.2^+^ vs CD90.2^−^). In some experiments, Rapamycin (Sigma-Aldrich, R8781; pre-diluted in DMSO) was diluted further in PBS (75 μg/kg) and injected i.p. at a volume of 0.2 ml for 14 days. See **Supplementary Figure 1C** for gating strategy.

### LCMV CL13 infection and determination of virus titers

LCMV CL13 were propagated and titers were determined as previously described (5). One kidney per LCMV CL13 infected-mice was homogenized (Cole-Parmer) in 1 ml of MEM-alpha media (Gibco) supplemented with 10% FBS. The virus titer of homogenized kidney fluid or serum was determined by plaque assay (5) and represented as plaque-forming units per ml (PFU/ml). To test the anti-viral activity of ASP1570, the plaque assay was performed in the presence or absence of ASP1570 (1 μM) with a fixed concentration of virus (1.4 × 10^7^ PFU/ml) added to the cell cultures. Mice were infected intravenously with 4 × 10^6^ PFU of LCMV CL13.

### *In vivo* systemic administration of DGKζ inhibitor ASP1570

For pharmacological inhibition of DGKζ activity *in vivo*, mice were administered p.o. once daily with 0.5% Methyl Cellulose (10 ml/kg; Wako Chemicals USA, Inc 13317815) or ASP1570 (3 mg/kg; Astellas Pharma, Inc; Patent: WO2021132422) for 3 Days before takedown. The drug was not given if the mouse was <80% of initial body weight on the day of administration.

### Phosphorylation flow cytometric assays

For *in vitro* phosphorylation assays, splenocytes were pretreated for 30 min with fluorescently labeled anti-CD8 and anti-CD44 antibodies and LIVE/DEAD Fixable near-IR Dead Cell Stain in the presence or absence of ASP1570 (1 μM). Splenocytes were washed and stimulated with anti-CD3 antibody (BD Biosciences) for the indicated times in the presence or absence of ASP1570 (1 μM). After stimulation, splenocytes were fixed in 2% paraformaldehyde and Perm Buffer III (BD Biosciences) and stained for anti-pERK and anti-pS6 intracellularly. Plots were gated on CD44^lo^CD8^+^ T cells. All fluorochrome-conjugated antibodies are listed in **Table S1**.

### Statistical analysis

Statistical tests for flow-cytometry data were performed using GraphPad Prism software. A P value of <0.05 was considered significant in these analyses. A Student t-test (two-tailed) was used for comparisons between two independent conditions. A paired Student t-test was used when the samples being compared originated from the same mouse. Log-rank test was used to determine significance of P value of <0.05.

## Results

### DGKζ-deficient LCMV-specific CD8^+^ T cells display increased effector differentiation during LCMV CL13 infection

**Figure 1:**
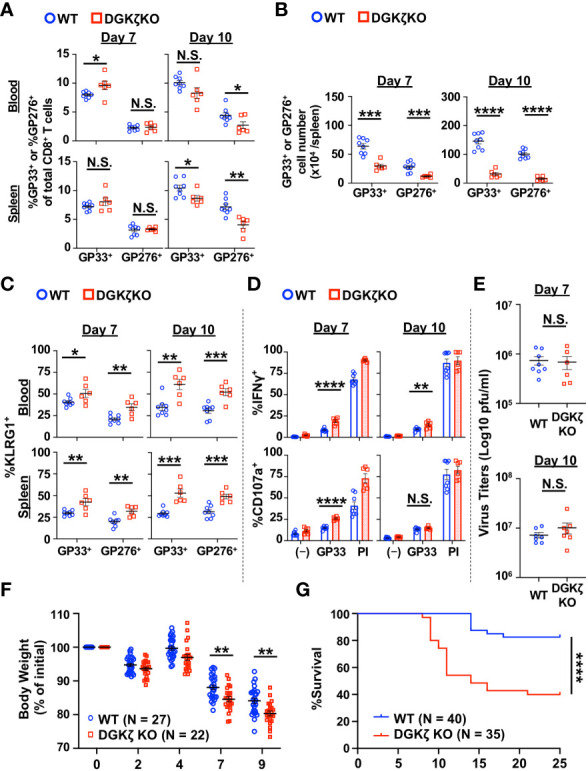
LCMV-specific T cells from DGKζ KO mice show increased activation after LCMV CL 13 infection. **(A)** The proportion and **(B)** absolute number (spleen only) of CD8^+^Tetramer^+^ (GP33^+^ or GP276^+^) cells and the **(C)** fraction of KLRG1^+^ cells of CD8^+^Tetramer^+^ cells were quantified in the blood and spleen of WT and DGKζ KO mice infected with LCMV CL 13 on Days 7 and 10 post infection. **(D)** The fraction of CD8^+^ T cells expressing IFNγ or CD107a was quantified in spleen cells isolated from LCMV CL13-infected WT and DGKζ KO mice on Days 7 or 10 post infection and restimulated with GP33 peptide or PMA and ionomycin (PI). **(E)** Virus titers in kidney of LCMV CL13-infected WT and DGKζ KO mice on Days 7 or 10 post infection. Data are represented as mean ± SEM of N=6-8 mice/group pooled from 2 independent experiments. **(F)** Body weight (N=22-27 mice/group from 4 independent experiments) and **(G)** survival (N=35-40 mice/group from 7 independent experiments) of WT and DGKζ KO mice infected with LCMV CL13. N.S. = not significant, *p<0.05, **P<0.01, ***p<0.001, ****p<0.0001 by Student t-test **(A–F)** or Log rank (Mantel Cox) test and Gehan-Breslow-Wilcoxon test **(G)**.

To test the role of DAG-mediated signaling in T cell activation during the acute phase of chronic viral infection, we infected WT and DGKζ KO mice with LCMV CL13 and examined the phenotype and function of LCMV-specific T cells against two immunodominant epitopes (GP33 and GP276 peptides) at Days 7 and 10 post infection. At Day 7 post infection, other than a slight increase in the fraction of GP33-specific CD8^+^ T cells (GP33-tetramer^+^) in the blood, the fraction of LCMV-specific T cells was unchanged in the spleen and blood of DGKζ KO compared to WT mice (**Figure 1A**). In contrast, at Day 10 post infection, the fraction of LCMV-specific CD8^+^ T cells was decreased in DGKζ KO compared to WT mice (**Figure 1A**). Moreover, owing to an overall decrease in splenocyte count in LMCV CL13-infected DGKζ KO mice (**Supplementary Figure 2**), the absolute number of LCMV-specific CD8^+^ T cells was significantly decreased at both Days 7 and 10 post infection in DGKζ KO compared to WT mice (**Figure 1B**). Despite the overall decreased number, the fraction of LCMV-specific CD8^+^ T cells expressing the effector differentiation marker, KLRG1 was significantly increased at both Days 7 and 10 post infection in DGKζ KO compared to WT mice (**Figure 1C**). Functionally, an increased fraction of splenic CD8^+^ T cells from DGKζ KO compared to WT mice were capable of degranulating (CD107a^+^) and producing IFNγ upon GP33 peptide restimulation ex vivo (**Figure 1D**). However, there was no difference in the virus titer of DGKζ KO and WT mice at either Day 7 or 10 post infection (**Figure 1E**, **Supplementary Figure 3**). These results suggested that DGKζ KO LCMV-specific CD8^+^ T cells showed increased effector differentiation and functional capacity but potentially impeded expansion and/or survival. Analysis of T cells beyond Day 10 was not performed due to significantly exacerbated weight loss and mortality of LCMV CL13-infected DGKζ KO mice (**Figures 1F, G**).

### DGKζ KO LCMV-specific CD8+ T cells display early proliferation and effector differentiation followed by abrupt collapse during LCMV CL13 infection

**Figure 2:**
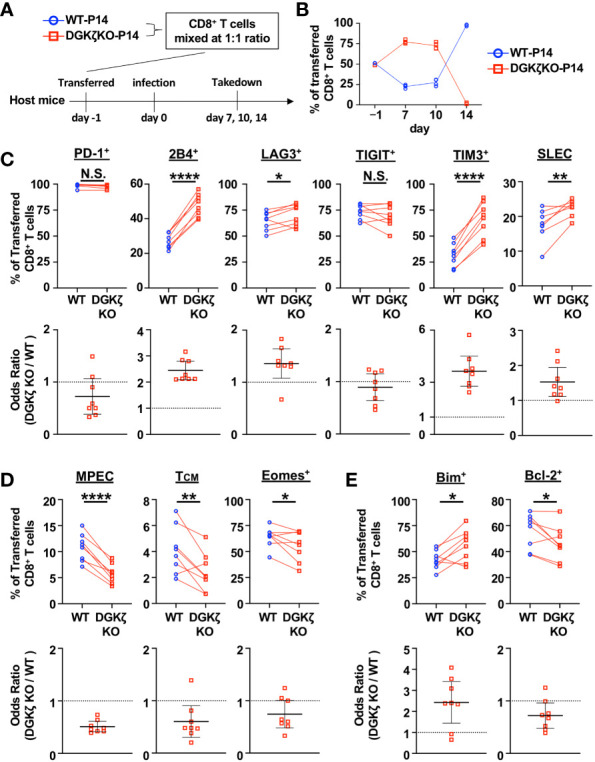
DGKζ-deficient LCMV-specific T cells display increased early effector differentiation followed by abrupt collapse in cell numbers. **(A)** CD8^+^ T cells from WT-P14 (CD45.1^+^) and DGKζ KO-P14 (CD45.2^+^) mice were mixed at a 1:1 ratio and adoptively transferred into Thy1.1^+^ WT host mice 1 day before infection with LCMV CL13. **(B)** The fraction of WT and DGKζ KO CD8^+^ T cells of all adoptively transferred P14 T cells was quantified over time in the spleen. Data are plotted as mean of N=4 mice/group per time point. One representative of 2 independent experiments is shown. **(C)** The fraction and odds ratio of splenic WT and DGKζ KO CD8^+^ T cells expressing PD-1, 2B4, LAG3, TIM3, TIGIT, or with a SLEC phenotype (KLRG1^+^CD127), **(D)** expressing a MPEC or T_CM_ phenotype or Eomes, or **(E)** expressing Bim or Bcl-2 were quantified at Day 10 post LCMV CL13 infection. Data from N=6-8 mice/group pooled from 2 independent experiments is shown. N.S. = not significant, *p<0.05, **P<0.01, ****p<0.0001 by paired Student t-test.

To test the impact of DGKζ deficiency on LCMV-specific CD8^+^ T cells beyond Day 10, we examined the activity of DGKζ KO T cells on a fixed LCMV GP33 peptide-specific TCR background (P14 TCR transgenic mice). This allowed us to directly compare WT and DGKζ KO T cells in the same environment and avoided confounding issues associated with altered TCR selection in the thymus of DGKζ KO mice. Naïve CD8^+^ T cells from congenically disparate (CD45.2^+^ vs CD45.1^+^) WT P14 and DGKζ KO P14 mice were mixed at a 1:1 ratio, adoptively transferred into WT mice (Thy1.1^+^), and subsequently infected with LCMV CL13 (**Figure 2A**). At Days 7 and 10 post infection, DGKζ KO P14 CD8^+^ T cells out-competed co-transferred WT P14 CD8^+^ T cells (**Figure 2B**), suggesting that DGKζ deficiency afforded an expansion advantage. Moreover, the fraction of inhibitory receptor-expressing cells and short-lived effector cells (SLEC; KLRG1^+^CD127^−^) were increased in DGKζ KO P14 CD8^+^ T cells compared to WT P14 CD8^+^ T cells (**Figure 2C**, **Supplementary Figures 4A, B**), suggesting increased effector cell differentiation. Almost all P14 CD8^+^ T cells of either WT or DGKζ KO origin degranulated and >70% produced IFNγ upon restimulation with GP33 peptide (**Supplementary Figure 5A**). A statistically significant increase in the fraction IFNγ-producing of DGKζ KO P14 CD8^+^ T cells compared to WT was seen on Days 7 and 10 post infection (**Supplementary Figure 5A**). In contrast to the increased SLEC phenotype, a smaller fraction of DGKζ KO P14 CD8^+^ T cells displayed a memory precursor effector cell (MPEC; KLRG1^−^CD127^+^) or central memory cell (T_CM_; CD62L^+^CD127^+^) phenotype. Additionally, fewer DGKζ KO P14 CD8^+^ cells expressed the memory-associated transcription factor Eomesodermin^+^ (Eomes^+^) phenotype (**Figure 2D**, **Supplementary Figure 4B**). Interestingly, an abrupt disappearance of DGKζ KO P14 CD8^+^ T cells was seen on Day 14 (**Figure 2B**, **Supplementary Figure 3B**), suggesting that DGKζ KO P14 CD8^+^ T cells underwent cell death. Consistent with these data, we found that the sudden crash in DGKζ KO P14 CD8^+^ T cells was preceded by a larger fraction of cells expressing the pro-apoptotic molecule Bim and fewer expressing the anti-apoptotic molecule Bcl-2 at Day 10 post infection (**Figure 2E**, **Supplementary Figure 4B**). Together, these data suggest that enhanced DAG-mediated signaling in LCMV-specific CD8^+^ T cells leads to rapid early expansion and effector differentiation with decreased proportion of cells expressing memory markers, which subsequently leads to disappearance of these cells.

### The selective activation of ERK enhances CD8^+^ T cell activation and decreases virus titers during LCMV CL13 infection

**Figure 3:**
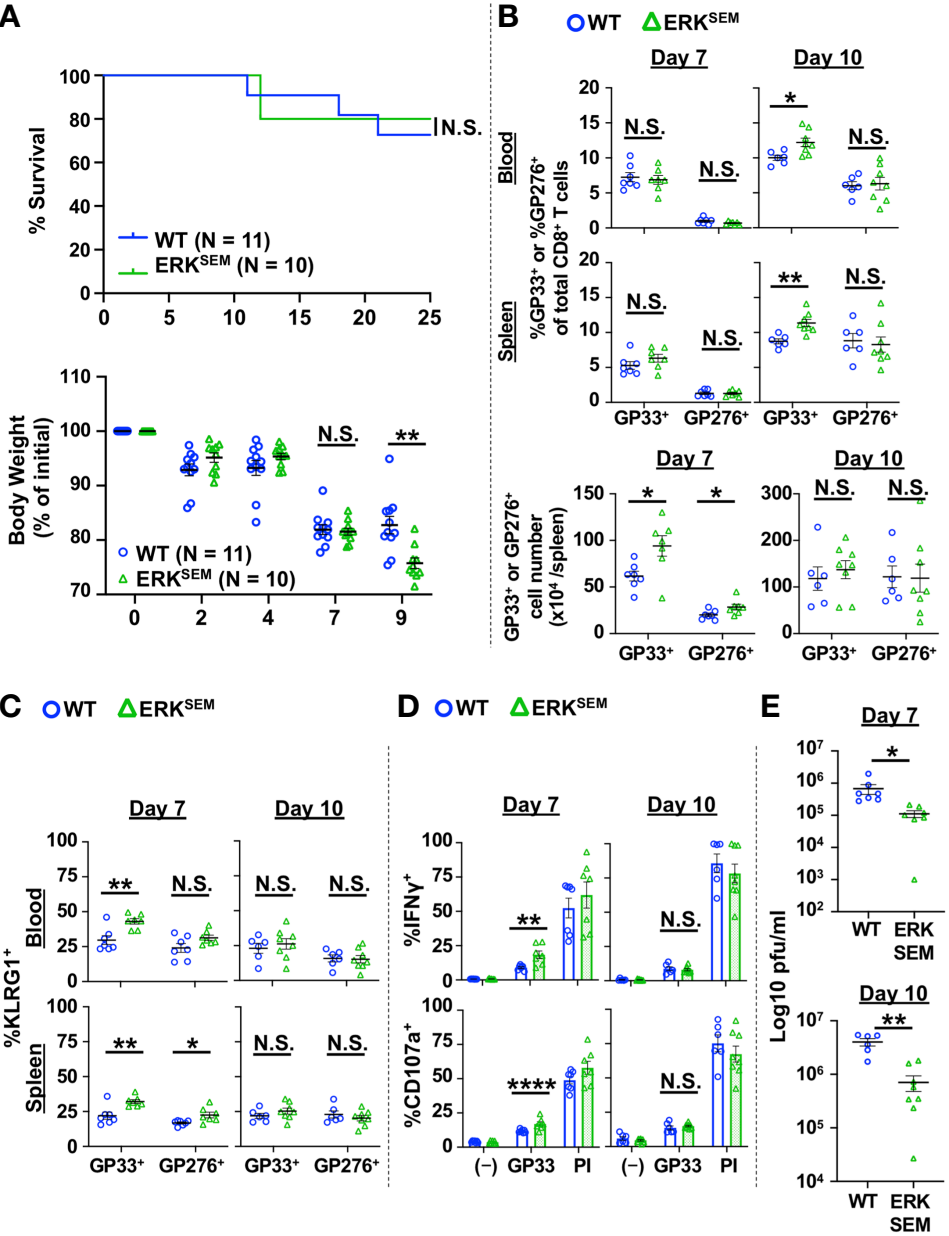
ERK^SEM^ mice show increased LCMV-specific CD8^+^ T cell activation and reduced virus titers after LCMV clone 13 infection. **(A)** Survival and body weight of WT and ERK^SEM^ mice infected with LCMV CL13. N=10-11 mice/group from 2 independent experiments. **(B)** The proportion and absolute number (spleen only) of CD8^+^Tetramer^+^ (GP33^+^ or GP276^+^) cells and the **(C)** fraction of KLRG1^+^ cells of CD8^+^Tetramer^+^ cells were quantified in the blood and spleen of WT and ERK^SEM^ mice infected with LCMV CL 13 on Days 7 and 10 post infection. **(D)** The fraction of CD8^+^ T cells expressing IFNγ or CD107a was quantified in spleen cells isolated from LCMV CL13-infected WT and ERK^SEM^ mice on Days 7 or 10 post infection and restimulated with GP33 peptide or PMA and ionomycin (PI). **(E)** Virus titers in kidney of LCMV CL13-infected WT and ERK^SEM^ mice on Days 7 or 10 post infection. Data are represented as mean ± SEM of N=6-8 mice/group pooled from 2 independent experiments. N.S. = not significant, *p<0.05, **P<0.01, ****p<0.0001 by Log rank (Mantel Cox) test and Gehan-Breslow-Wilcoxon test **(A)** or Student t-test **(B–E)**.

ERK activation is an important signaling event that is downstream of DAG. To test whether the effect of DAG-mediated signaling on T cells was mediated by ERK activation, we utilized mice with a gain-of-function of ERK (sevenmaker mutation; ERK^SEM^) in T cells. This mutation makes ERK more resistant to dephosphorylation, leading to prolonged activation of ERK (22, 24). The T cell compartment of ERK-SEM mice is largely intact, having a slight reduction in total number of thymocytes but normal numbers of lymph node T cells (22). LCMV CL13 infection of ERK^SEM^ mice did not cause increased mortality as seen in DGKζ KO mice, although some exacerbation of weight loss was seen at Day 9 post infection (**Figure 3A**). The fraction and number of LCMV-specific T cells of WT and ERK^SEM^ mice were largely similar at Days 7 and 10 post infection, although slight increases were seen in the absolute number of LCMV-specific CD8^+^ T cells at Day 7 and the fraction of GP33-specific CD8^+^ T cells at Day 10 post infection in ERK^SEM^ mice (**Figure 3B**). Compared to DGKζ KO mice, there was only a modest increase in the fraction of KLRG1^+^ cells, whereby an increase was only seen in GP33-specific CD8^+^ T cells in ERK^SEM^ mice at Day 7 post infection (**Figure 3C**). Similarly, an increased fraction of degranulating and IFNγ-producing CD8^+^ T cells upon LCMV GP33 peptide restimulation was seen in ERK^SEM^ mice at Day 7 but not at Day 10 post infection (**Figure 3D**). Virus titers were significantly decreased in the kidneys of ERK^SEM^ mice at both Days 7 and 10 post infection (**Figure 3E**). The virus load in serum was ~2 log lower than kidney, but no difference was seen between WT and ERK^SEM^ mice (**Supplementary Figure 3B**). These results suggested that the anti-viral activity of ERK^SEM^ T cells was enhanced, but the activation phenotype may not have been pronounced due to decreased viral titers.

### The selective activation of ERK promotes proliferation, survival, and memory phenotype of LCMV-specific CD8^+^ T cells during LCMV CL13 infection

**Figure 4:**
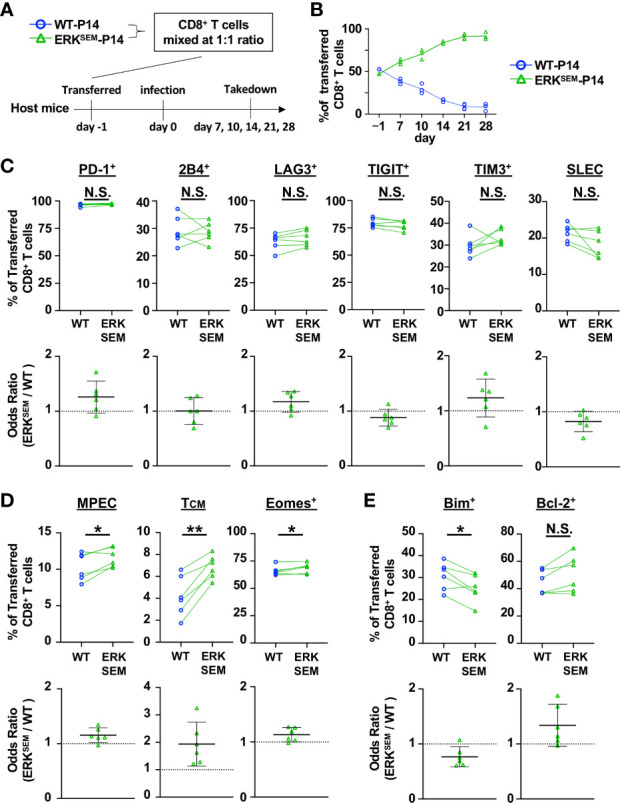
ERK^SEM^ LCMV-specific T cells display increased proliferation, survival, and memory phenotype. **(A)** CD8^+^ T cells from WT-P14 (CD45.1^+^) and ERK^SEM^-P14 (CD45.2^+^) mice were mixed at a 1:1 ratio and adoptively transferred into Thy1.1^+^ WT host mice 1 day before infection with LCMV CL13. **(B)** The fraction of WT and ERK^SEM^ T cells of all adoptively transferred P14 CD8^+^ T cells was quantified over time in the spleen. Data are plotted as mean of N=3-4 mice/group per time point. One representative of 2 independent experiments is shown. **(C)** The fraction and odds ratio of splenic WT and ERK^SEM^ CD8^+^ T cells expressing PD-1, 2B4, LAG3, TIM3, TIGIT, or with a SLEC phenotype (KLRG1^+^CD127), **(D)** expressing a MPEC or T_CM_ phenotype or Eomes, or **(E)** expressing Bim or Bcl-2 were quantified at Day 10 post LCMV CL13 infection. Data from N=6 mice/group pooled from 2 independent experiments are shown. N.S. = not significant, *p<0.05, **P<0.01 by paired Student t-test.

Since ERK^SEM^ mice had decreased viral titers relative to WT mice, next crossed ERK^SEM^ mice to a P14 background to enable a more rigorous comparison of T cell differentiation in a setting with normalized viral burden. To this end, naïve CD8^+^ T cells from CD45.2^+^ ERK^SEM^ P14 mice and CD45.1^+^ WT P14 mice were mixed in a 1:1 ratio and adoptively transferred into Thy1.1^+^ mice and infected with LCMV CL13 (**Figure 4A**). Similar to DGKζ KO P14 CD8^+^ T cells (**Figure 2B**), ERK^SEM^ P14 CD8^+^ T cells outcompeted WT P14 T cells at Days 7 and 10 (**Figure 4B**). Moreover, almost all P14 CD8^+^ T cells of either WT or ERK^SEM^ origin degranulated and >60% produced IFNγ upon restimulation with GP33 peptide (**Supplementary Figure 5B**). A statistically significant increase in the fraction IFNγ-producing of ERK^SEM^ P14 CD8^+^ T cells compared to WT was seen on Day 10 post infection (**Supplementary Figure 5B**). Interestingly, while DGKζ KO P14 CD8^+^ T cells disappeared at Day 14 (**Figure 2B**), ERK^SEM^ P14 CD8^+^ T cells continued to increase even after Day 14 post infection, representing >90% of P14 T cells by Day 28 (**Figure 4B**). Although the fraction of inhibitory receptor-expressing cells and SLEC phenotype was unchanged (**Figure 4C**, **Supplementary Figures 6A, B**), the proportion of ERK^SEM^ MPEC, T_CM_, and Eomes^+^ P14 cells were increased on Day 10 post infection (**Figure 4D**, **Supplementary Figure 6B**). Consistent with their increased survival, the proportion of ERK^SEM^ P14 CD8^+^ T cells expressing Bim was significantly lower with a trend towards an increased proportion of Bcl-2-expressing cells (**Figure 4E**, **Supplementary Figure 5B**). These data suggest that the selective enhancement of ERK does not phenocopy DGKζ deficiency. Rather, isolated ERK augmentation leads to an increased proportion of CD8^+^ T cells with a memory phenotype and to increased proliferation and survival of CD8^+^ T cells without a loss in effector differentiation and anti-viral activity.

### Short term inhibition of DGKζ increases LCMV-specific CD8^+^ T cell activation and lowers virus titers in the acute and chronic phases of LCMV CL13 infection

**Figure 5:**
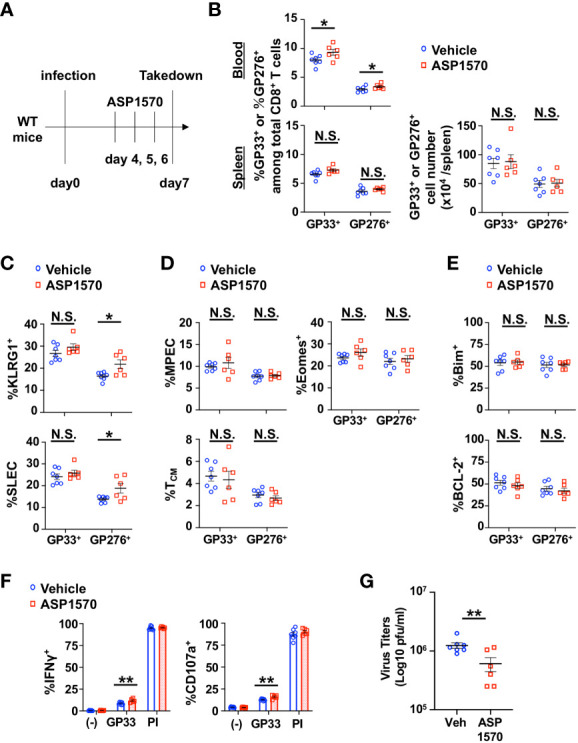
Short-term inhibition of DGKζ increases LCMV-specific CD8^+^ T cell activation and decreases virus titers in the acute phase of LCMV CL 13 infection. **(A)** WT mice were infected with LCMV CL13 and treated with either vehicle or ASP1570 on Days 4, 5, and 6 post infection. **(B)** The proportion (blood and spleen) and absolute number of CD8^+^Tetramer^+^ (GP33^+^ or GP276^+^) cells and the fraction of **(C)** KLRG1^+^, SLEC phenotype cells, **(D)** Bim^+^ cells, **(E)** MPEC and T_CM_ phenotype cells of CD8^+^Tetramer^+^ cells were quantified in the spleen of vehicle and ASP1570-treated mice on Day 7 post LCMV CL13 infection. **(F)** The fraction of CD8^+^ T cells expressing IFNγ or CD107a was quantified in spleen cells isolated from vehicle and ASP1570-treated mice on Day 7 post LCMV CL13 infection and restimulated with GP33 peptide or PMA and ionomycin (PI). **(G)** Virus titers in kidney of vehicle and ASP1570-treated mice on Day 7 post LCMV CL13 infection. Data from N=6-7 mice/group pooled from 2 independent experiments are shown. N.S. = not significant, *p<0.05, **P<0.01 by Student t-test.

Since the constitutive lack of DGKζ in T cells led to the disappearance of LCMV-specific CD8^+^ T cells beyond Day 14 post LMCV CL13 infection, we could not test how DGKζ deficiency affected T cell exhaustion in the chronic phase of LCMV CL13 infection. Thus, we employed a recently developed highly potent DGKζ-selective inhibitor (ASP1570) to temporally inhibit DGKζ function. We first tested the effect of ASP1570 in the acute phase of LCMV CL13 infection. ASP1570 or vehicle was administrated for 3 Days before takedown at Day 7 post infection (**Figure 5A**). Short term ASP1570 treatment early in infection led to a higher proportion of LCMV-specific CD8^+^ T cells in blood but not in the spleen at Day 7 (**Figure 5B**). The proportion of GP276-specific but not GP33-specific CD8^+^ T cells expressing KLRG1 and displaying a SLEC phenotype was increased in ASP1570-treated mice (**Figure 5C**). However, no differences were seen in fraction of LCMV-specific CD8^+^ T cells expressing Eomes or displaying a T_CM_ or MPEC phenotype (**Figure 5D**). Moreover, the fraction of LCMV-specific CD8^+^ T cells expressing Bim or BCL-2 remained unchanged (**Figure 5E**). Ex vivo restimulation with GP33 peptide revealed an increased fraction of degranulating and IFNγ-producing CD8^+^ T cells (**Figure 5F**). Interestingly, there was a small but statistically significant decrease in viral titers in the kidney but not in the serum with ASP1570 treatment (**Figure 5G**, **Supplementary Figure 3C**), which could potentially explain some of the differences in T cell phenotype seen between the DGKζ KO mice and DGKζ inhibitor treatment. Of note, ASP1570 did not display any direct anti-viral effect against LCMV CL13 (**Supplementary Figure 7**).

**Figure 6:**
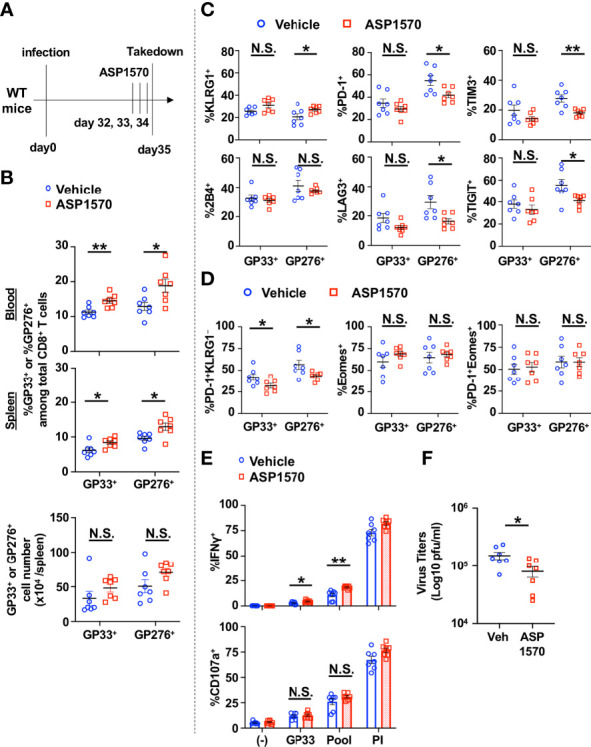
Short-term inhibition of DGKζ increases LCMV-specific CD8^+^ T cell activation and decreases virus titers in the chronic phase of LCMV CL 13 infection. **(A)** WT mice were infected with LCMV CL13 and treated with either vehicle or ASP1570 on Days 32, 33, and 34 post infection. **(B)** The proportion (blood and spleen) and absolute number of CD8^+^Tetramer^+^ (GP33^+^ or GP276^+^) cells and the fraction of **(C)** KLRG1^+^, PD-1^+^, 2B4^+^, LAG3^+^, TIGIT^+^, TIM3^+^, or **(D)** PD-1^+^KLRG1^−^, Eomes^+^, PD-1 KLRG1^+^Eomes^+^ cells of CD8^+^Tetramer^+^ cells were quantified in the spleen of vehicle and ASP1570-treated mice on Day 35 post LCMV CL13 infection. **(E)** The fraction of CD8^+^ T cells expressing IFNγ or CD107a was quantified in spleen cells isolated from vehicle and ASP1570 -treated mice on Day 35 post LCMV CL13 infection and restimulated with GP33 peptide or PMA and ionomycin (PI). **(F)** Virus titers in kidney of vehicle and ASP1570 -treated mice on Day 35 post LCMV CL13 infection. Data from N=7 mice/group pooled from 2 independent experiments are shown. N.S. = not significant, *p<0.05, **P<0.01 by Student t-test.

We next tested the effect of short term DGKζ inhibition in the chronic phase of LCMV CL13 infection. ASP1570 or vehicle was administrated for 3 Days before takedown at Day 35 post infection (**Figure 6A**). A significant increase in the proportion and a trend towards an increase in absolute number of LCMV-specific CD8^+^ T cells were observed after ASP1570 treatment (**Figure 6B**). The fraction of GP276-specific CD8^+^ T cells expressing KLRG1 was increased and the proportion of GP276-specific CD8^+^ T cells expressing some of the inhibitory receptors (PD-1, LAG3, TIM3, TIGIT) was decreased after ASP1570 treatment (**Figure 6C**). In the chronic phase of LCMV CL13 infection, exhausted T cells express low levels of KLRG1 (25) and the co-expression of Eomes and PD-1 marks terminally exhausted T cells (26). While there was a decrease in the proportion of exhausted (PD-1^+^KLRG1^−^) LCMV-specific CD8^+^ T cells, the fraction of the most terminally exhausted T cells (PD-1^+^Eomes^+^) was not different (**Figure 6D**). To test for T cell function, restimulation with GP33 peptide and a LCMV peptide pool was used (23), since T cells specific for other LCMV-derived peptides are increased in the later phases of LCMV CL13 infection. Although degranulation was unchanged, the fraction of IFNγ-producing CD8^+^ T cells was increased in ASP1570-treated mice at Day 35 post infection compared to vehicle-treated mice (**Figure 6E**). Importantly, short term ASP1570 treatment reduced virus titers at Day 35 post infection (**Figure 6F**). These results suggest that short-term DGKζ inhibition might provide some benefit in reinvigorating exhausted T cells in the setting of chronic viral antigen exposure.

### ERK^SEM^ mice display decreased LCMV-specific CD8^+^ T cell exhaustion and lower virus titers in the chronic phase of LCMV CL13 infection

**Figure 7:**
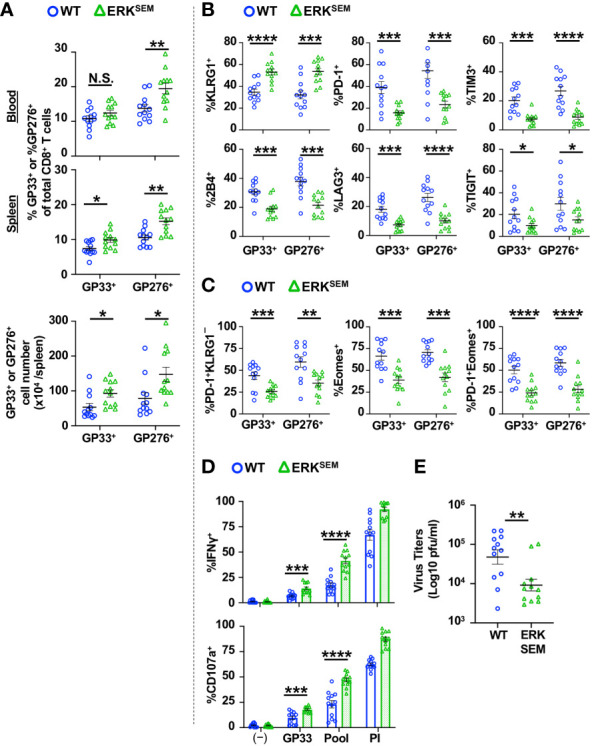
ERK^SEM^ mice have fewer exhausted LCMV-specific CD8^+^ T cells and lower virus titers in the chronic phase of LCMV CL 13 infection. **(A)** The proportion (blood and spleen) and absolute number of CD8^+^Tetramer^+^ (GP33^+^ or GP276^+^) cells and the fraction of **(B)** KLRG1^+^, PD-1^+^, TIM3^+^, 2B4^+^, LAG3^+^, TIGIT^+^ or **(C)** PD-1^+^KLRG1^−^, Eomes^+^, PD-1 KLRG1^+^Eomes^+^ cells of CD8^+^Tetramer^+^ cells were quantified in the spleen of WT and ERK^SEM^ mice on Day 35 post LCMV CL13 infection. **(D)** The fraction of CD8^+^ T cells expressing IFNγ or CD107a was quantified in spleen cells isolated from WT and ERK^SEM^ mice on Day 35 post LCMV CL13 infection and restimulated with GP33 peptide or PMA and ionomycin (PI). **(E)** Virus titers in kidney of WT and ERK^SEM^ mice on Day 35 post LCMV CL13 infection. Data from N=12 mice/group pooled from 3 independent experiments are shown. N.S. = not significant, *p<0.05, **P<0.01, ***p<0.001, ****p<0.0001 by Student t-test.

We next sought to test the impact of the selective activation of ERK in the chronic phase of LCMV CL13 infection. LCMV CL13-infected ERK^SEM^ mice harbored a higher proportion and absolute number of LCMV-specific CD8^+^ T cells at Day 35 post infection compared to WT mice (**Figure 7A**). The fraction of LCMV-specific CD8^+^ T cells expressing KLRG1 was higher and those expressing inhibitory receptors (**Figure 7B**) were markedly lower in ERK^SEM^ compared to WT mice. Moreover, the proportion of exhausted (PD-1^+^KLRG1^−^) and the most terminally exhausted LCMV-specific CD8^+^ T cells (PD-1^+^ Eomes^+^) was significantly lower in ERK^SEM^ compared to WT mice (**Figure 7C**). Functionally, the fraction of CD8^+^ T cells degranulating and producing IFNγ was higher after LCMV peptide restimulation (**Figure 7D**). Moreover, ERK^SEM^ mice displayed reduced virus titers (**Figure 7E**). Thus, the selective activation of ERK pathway leads to favorable anti-viral T cell responses that extends into the chronic phase with less evidence of T cell exhaustion.

### mTOR inhibition blocks cell death of DGKζ KO P14 T cells at Day 14 post-infection

**Figure 8:**
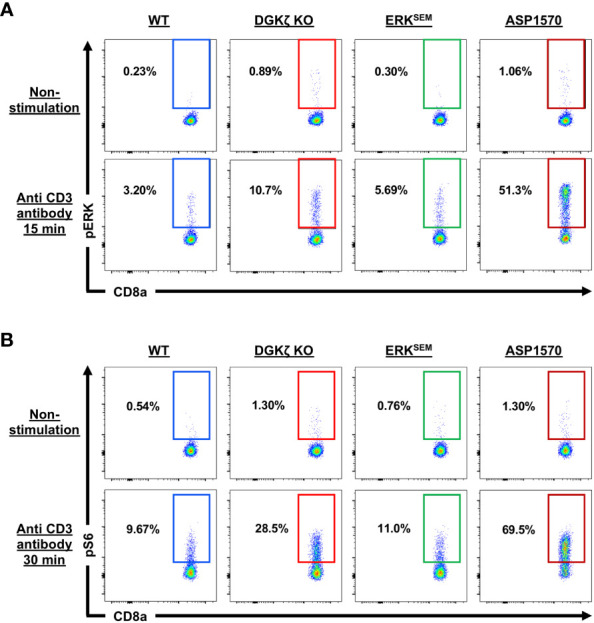
ERK and S6 phosphorylation in TCR-stimulated DGKζ KO, ERK^SEM^, and ASP1570-treated CD8^+^ T cells. **(A)** Representative flow cytometric plots of CD44^lo^ CD8^+^ T cells displaying pERK and **(B)** pS6 are shown in unstimulated and CD3-stimulated CD44^lo^ CD8^+^ T cells of the indicated genotype and treatment. One representative of 2 experiments is shown.

To examine the extent of ERK activation in DGKζ KO, ERK^SEM^, and ASP1570-treated CD8^+^ T cells, we quantified phospho-ERK (pERK) after TCR stimulation. We found that both DGKζ deficiency, ERK^SEM^ expression, and ASP1570 treatment augmented the fraction of CD8^+^ T cells expressing pERK (**Figure 8A**, **Supplementary Figure 8A**). The fraction of pERK^+^ ERK^SEM^ CD8^+^ T cells was less than DGKζ deficiency. This could be because the ERK^SEM^ mutation is located only in ERK2 and thus. Moreover, the ERK^SEM^ mutation delays ERK dephosphorylation but may not necessarily increase the probability of ERK phosphorylation. Consistent with this notion, the amount (MFI) of pERK in pERK^+^ CD8^+^ T cells was increased (**Supplementary Figure 8B**). Since DAG also stimulates the AKT/mTOR pathway, we next examined the phosphorylation of S6 (a surrogate readout of the AKT/mTOR pathway) in DGKζ KO, ERK^SEM^, and ASP1570-treated CD8^+^ T cells. We found that DGKζ deficiency and ASP1570 treatment augmented the fraction of CD8^+^ T cells expressing pERK, whereas this was not consistently seen in ERK^SEM^ CD8^+^ T cells (**Figure 8B**, **Supplementary Figure 8C**).

**Figure 9:**
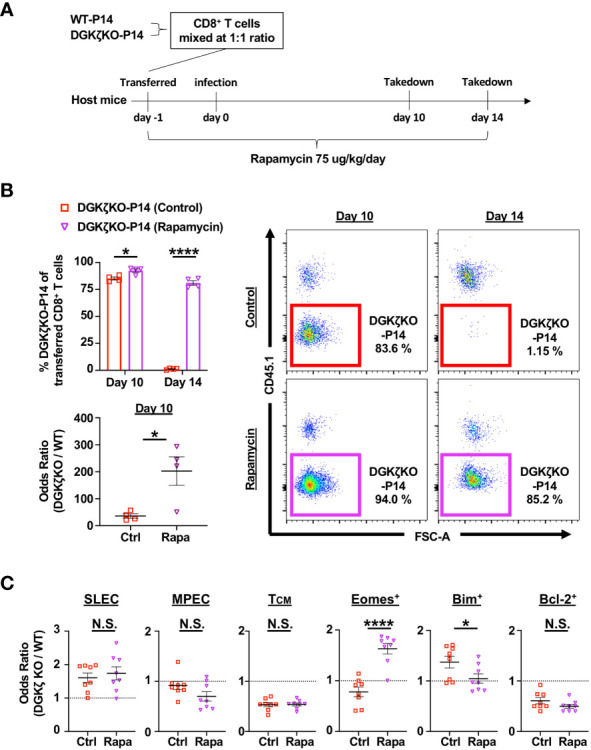
Rapamycin treatment rescues DGKζ KO CD8^+^ T cells from disappearing at Day 14 post infection. **(A)** CD8^+^ T cells from WT-P14 (CD45.1^+^) and DGKζ KO-P14 (CD45.2^+^) mice were mixed at a 1:1 ratio and adoptively transferred into Thy1.1^+^ WT host mice 1 day before infection with LCMV CL13. One group of mice was treated with vehicle and another with rapamycin. **(B)** The fraction of WT and DGKζ KO CD8^+^ T cells of all adoptively transferred P14 T cells was quantified at Days 10 and 14 post infection in the spleen. The odds ratio of splenic WT vs. DGKζ KO CD8^+^ T cells was compared in mice treated with vehicle vs. rapamycin at Day 10 post infection. One representative of 2 independent experiments is shown. **(C)** The odds ratio of splenic WT and DGKζ KO CD8^+^ T cells expressing a SLEC phenotype (KLRG1^+^CD127^−^), an MPEC or T_CM_ phenotype, Eomes, Bim, or Bcl-2 were quantified at Day 10 post LCMV CL13 infection and compared between vehicle and rapamycin treatment. Data from N=8 mice/group pooled from 2 independent experiments is shown. N.S. = not significant, *p<0.05, or ****p<0.0001 by Student t-test.

Blocking mTOR activity promotes the expression of Eomes, Bcl-2, and CD62L, which leads to increased memory generation and KLRG1^low^ cells (27). Thus, we wondered whether the enhanced mTOR signal was responsible for the difference in survival seen in DGKζ KO vs. ERK^SEM^ CD8^+^ T cells. To test this possibility, WT CD8^+^ P14 and DGKζ KO CD8^+^ P14 mice were mixed at a 1:1 ratio, adoptively transferred into WT mice (Thy1.1^+^), and subsequently infected with LCMV CL13 and treated with vehicle or rapamycin daily (**Figure 9A**). We found that treatment with rapamycin significantly augmented the survival advantage of DGKζ KO P14 CD8^+^ T cells at Day 10 post infection (**Figure 9B**). Strikingly, at Day 14 post infection when DGKζ KO P14 CD8^+^ T cells disappear in vehicle-treated mice, rapamycin-treated mice still show a significant survival advantage of DGKζ KO P14 CD8^+^ T cells (**Figure 9B**). The survival of DGKζ KO P14 CD8^+^ T cells at Day 14 was preceded by a decrease CD8^+^ T cells expressing Bim and an increase in cells expressing Eomes with rapamycin compared to vehicle treatment at Day 10 post infection (**Figure 9C**). However, DGKζ KO P14 CD8^+^ T cells expressing a SLEC, MPEC, or T_CM_ phenotype, or Bcl-2 was unaltered by rapamycin treatment at Day 10 post infection (**Figure 9C**). These results suggest that increased mTOR signaling in DGKζ KO might explain why the survival of CD8^+^ T cells is different from the selective enhancement of ERK by the ERK^SEM^ mutation.

## Discussion

In this study, we sought to test the impact of enhanced DAG-mediated signaling in T cells during chronic viral infection. As expected, we found that augmenting DAG signaling by DGKζ deficiency enhances antigen-specific CD8^+^ T cell responses during the acute phase of LCMV CL13 infection. Compared to WT mice, DGK ζ KO mice exhibited a higher fraction of virus-specific CD8^+^ T cells, more of which expressed KLRG1 at Day 7 post infection. Moreover, a larger fraction of DGKζ KO CD8^+^ T cells degranulated and produced IFNγ upon LCMV peptide restimulation compared to WT CD8^+^ T cells. These results were similar to those seen in prior studies showing enhanced IFNγ production after stimulation with LCMV-specific peptides GP33, NP396, and GP276 after LCMV Armstrong infection (28, 29).

One caveat with our studies was that a substantial fraction of DGK ζ KO mice succumbed to viral infection after Day 10 post infection, making it difficult to study how DAG-mediated signaling affected T cell responses later in infection. Thus, we resorted to an adoptive transfer model whereby DGKζ KO LCMV GP33-specific TCR transgenic T cells (P14 T cells) could be tracked in competition with WT P14 T cells after LCMV CL13 infection. This also allowed us to negate the effects that DGKζ deficiency might have on developing T cells during TCR selection in the thymus. Consistent with the notion that DGKζ enhances antigen-specific CD8^+^ T cell responses, DGKζ KO P14 T cells outcompeted WT P14 T cells at Days 7 and 10 post LCMV CL13 infection. Unexpectedly, however, DGKζ KO P14 T cells were barely detectable by Day 14 post infection and beyond, suggesting that DGKζ KO P14 underwent cell death caused by excessive TCR signaling. In agreement with this notion, an increased fraction of DGKζ KO P14 T cells expressing Bim and a decreased fraction of DGKζ KO P14 T cells expressing Bcl2 were observed compared to WT P14 T cells.

Many of the phenotypes that are seen in DGKζ KO mice are secondary to enhanced activation of the ERK pathway. As such, T cells from ERK^SEM^ mice phenocopy those from DGK ζ KO mice in many aspects (20, 21, 30). Thus, we predicted that ERK^SEM^ T cells would also behave similarly to DGKζ KO T cells during LCMV CL13 infection. Indeed, ERK^SEM^ mice displayed an increased fraction of LCMV-specific KLRG1^+^ CD8^+^ T cells compared to WT mice. Moreover, a larger fraction of T cells from ERK^SEM^ compared to WT mice degranulated and produced IFNγ upon LCMV peptide restimulation. However, in contrast to DGKζ KO mice, ERK^SEM^ mice did not show increased susceptibility to LCMV CL13-induced mortality. Furthermore, the number of LCMV-specific T cells in ERK^SEM^ mice remained elevated beyond Day 7 post infection compared to WT mice. The enhanced survival and proliferation of ERK^SEM^ T cells was confirmed by competition experiments between ERK^SEM^ and WT P14 T cells during LCMV CL13 infection. Thus, T cells with selective activation of ERK appeared to have a survival advantage over WT and DGK ζ KO T cells. Accordingly, a smaller fraction of ERK^SEM^ P14 T cells expressed Bim and a larger fraction expressed Bcl-2 at Day 10 post infection. In addition, there was an increased fraction of ERK^SEM^ P14 T cells with an MPEC and T_CM_ phenotype. These results suggested that survival, proliferation, and differentiation to memory T cells was enhanced by selective enhancement of the ERK pathway.

The ERK signaling pathway has been previously associated with anti-apoptotic activity. Several kinases such as ERK2, MAPK, and CDK1 can phosphorylate the apoptotic initiator protease caspase-9, which inhibits caspase-9 to dampen the threshold for intrinsic apoptosis signals during the cell cycle (31, 32). Moreover, activation of the ERK pathway blocks the expression of Bim (31, 33–36), increases the transcription of the anti-apoptotic subfamily of Bcl-2 members (Bcl-2, MCL-1, Bcl-xL, Bcl-W, Bfl-1), and augments the ubiquitination and subsequent degradation of pro-apoptotic members (BAX, BAK), leading to increased cell survival (37–40). In a study using LCMV Armstrong infection, ERK1 and ERK2-deficient mice showed impaired proliferation and survival of LCMV-specific CD8^+^ T cells *in vivo*. The defective survival of ERK2-deficient CD8^+^ T cells was rescued by deletion of Bim *in vitro* (41).

ERK-dependent activation of pro-survival signals may be responsible for the increased memory T cell phenotype, as prevention of apoptosis by altering Bcl-2 or Bim expression correlate with the proliferation of memory T cells (42, 43). In contrast to ERK^SEM^, the fraction of DGK ζ KO P14 MPEC and T_CM_ phenotype were decreased compared to WT P14 T cells. However, in comparison to WT P14 T cells, the percentage of SLEC phenotype cells and inhibitory receptor-expressing cells was increased in DGKζ KO P14 T cells but not in ERK^SEM^ P14 T cells. These results suggest that DGKζ deficiency may promote the proliferation of antigen-specific CD8^+^ T cells mainly of SLEC phenotype, which are destined for apoptosis. A defect in DGKζ KO CD8^+^ T cells to establish long term memory was also seen in a study using LCMV Armstrong infection, whereby DGKζ KO LMCV-specific memory CD8^+^ T cells were reduced the number and exhibited impaired expansion after rechallenge (28).

**Figure 10:**
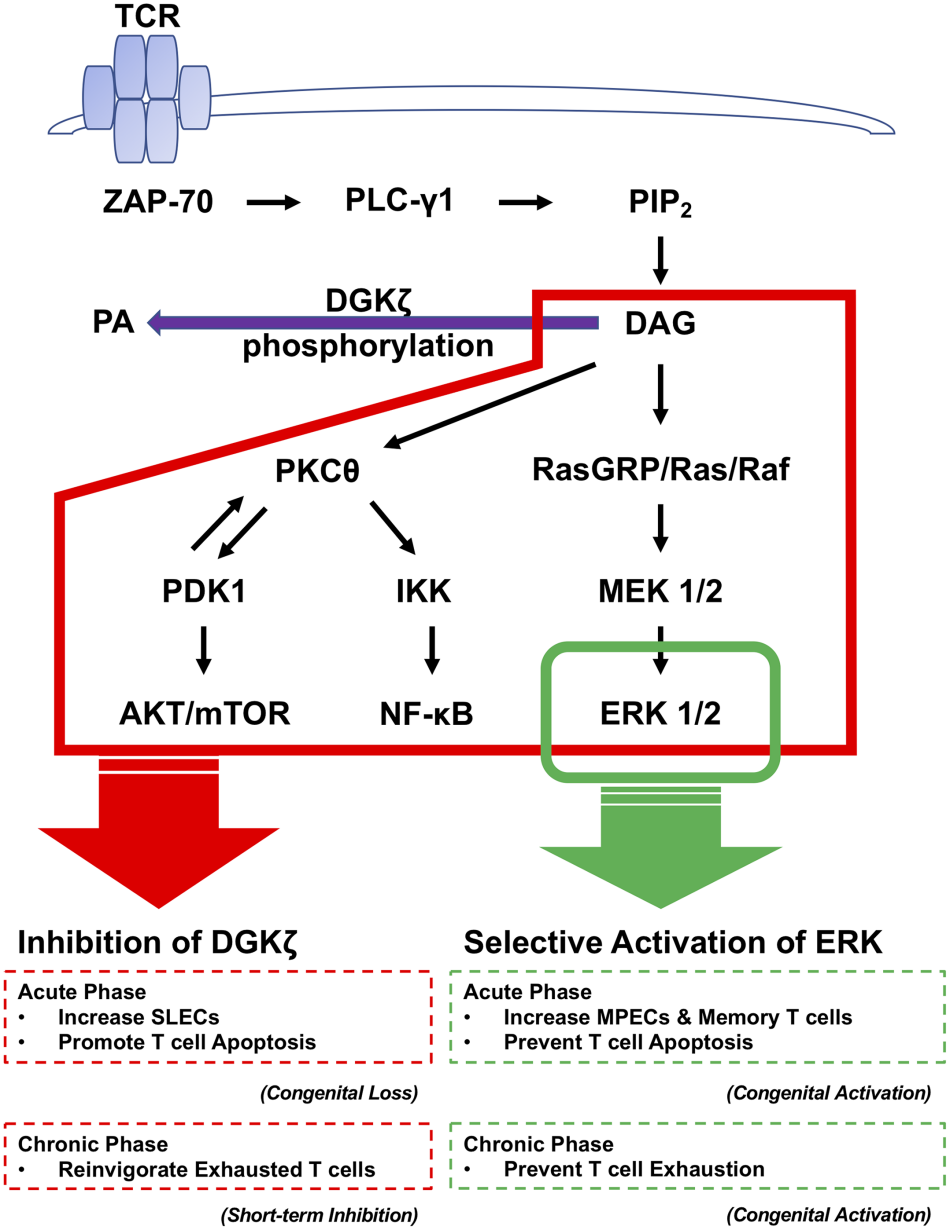
The contribution of DAG and ERK signaling pathways to T cell activation during the acute and chronic phase of LCMV CL13 infection. Engagement of the TCR activates a proximal signaling cascade that leads to the generation of DAG. DAG-mediated signaling is terminated by phosphorylation to PA by DGKζ. DAG induces signaling of the ERK pathway through activation of RasGRP. DAG also induces signaling through AKT/mTOR and NF-κB by activation of PKCθ. While the enhancement of ERK signaling increases memory phenotype T cells and prevents T cell apoptosis, the congenital loss of DAG DGKζ promotes effector differentiation and T cell apoptosis in the acute phase. In the chronic phase, both the enhancement of ERK signaling and short term pharmacological DGKζ blockade improve T cell exhaustion.

The difference in survival of ERK^SEM^ vs. DGKζ KO T cells suggest that DAG-dependent pathways that do not involve ERK might be directing cells down the SLEC differentiation pathway at the expense of memory formation. Other than ERK signaling, DAG activates NF-κB through PKCθ and the mTOR pathway through AKT (11, 12, 17, 18, 29, 44, 45). Therefore, there was a possibility that the difference between DGKζ KO and ERK^SEM^ was triggered by the NF-κB pathway and/or mTOR pathway. In general, the NF-κB pathway has been reported to prevent T cell apoptosis by inducing expression of several Bcl-2 family members and inhibiting the expression of Bim (46–48). In contrast, blocking mTOR activity promotes the expression of Eomes, Bcl-2, and CD62L, which leads to increased memory generation and KLRG1^low^ cells (27). In a study using LCMV Armstrong infection, treatment of mice with the mTOR inhibitor rapamycin enhanced not only the quantity but also the quality of LCMV-specific CD8^+^ T cells (49). Memory CD8^+^ T cells generated in the presence of rapamycin displayed a higher frequency of CD127^+^, CD62L^+^, Bcl-2^+^, and KLRG1^low^ cells compared to control mice (49). Rapamycin was effective during both the expansion and contraction phases of the T-cell response; during the expansion phase, it increased the number of memory precursors (CD127^high^ KLRG1^low^), and during the contraction phase (effector to memory transition) it accelerated the memory T-cell differentiation program (49). In our current study, we found that rapamycin treatment rescued the cell death of DGKζ KO CD8^+^ T cells seen during chronic viral infection. Thus, the increased activation of mTOR may be responsible for the decreased survival of virus-specific T cells observed in DGKζ KO mice compared to selective enhancement of ERK activation (**Figure 10**).

We do not know why the constitutive lack of DGKζ does not lead to decreased viral titers, despite increased activity of virus-specific CD8^+^ T cells. DGKζ KO mice display decreased splenocyte cell counts on Days 7 and 10 post LCMV CL13 infection. Consequently, this led to a corresponding decrease in the absolute number of virus-specific CD8^+^ T cells in LCMV CL13-infected DGKζ KO mice, which could have affected overall viral clearance. Although DGKζ KO mice did not cause a favorable anti-viral response, short term inhibition of DGKζ with ASP1570 showed some benefit in the anti-viral T cell response. Short term ASP1570 treatment during the acute phase of LCMV CL13 infection induced a higher percentage of LCMV-specific T cells with an MPEC and T_EM_ phenotype with more T cells degranulating and expressing IFNγ upon LCMV peptide restimulation. In addition, contrary to DGKζ KO mice, ASP1570 treatment reduced LCMV virus titers at Day 7 post infection. Still, similar to DGKζ KO mice, the fraction of LCMV-specific T cells expressing Bim was higher after administration of ASP1570.

Chronic infection with LCMV CL13 leads to CD8^+^ T cell exhaustion. One of the defining features of exhausted CD8^+^ T cells is high and persistent expression of inhibitory receptors including PD-1, LAG3, TIM3, 2B4, and TIGIT (7, 9). The presence of persisting viral antigen drives the continuous expression of inhibitory receptors, limiting the ability of anti-viral T cells to control the infection and enforcing their state of exhaustion (7, 9). Eomes is typically associated with memory CD8^+^ T cells following acute LCMV infection, but during chronic LCMV infection Eomes is linked to terminally exhausted cells with poor survival and proliferative abilities (9). Thus, PD-1^hi^ Eomes^hi^ and PD-1^hi^ KLRG1^lo^ cells are characteristic phenotypes of exhausted T cells (2, 8, 9, 50). Although we were unable to test how genetic deletion of DGKζ impacts the chronic phase of LCMV infection, we were able to test the role of ERK and short term DGKζ inhibition in the chronic phase. Both short term ASP1570 treatment mice and ERK^SEM^ mice demonstrated an increased percentage of LCMV-specific KLRG1^+^ CD8^+^ T cells accompanied by a smaller fraction of T cells expressing inhibitory receptors. In addition, ERK^SEM^ mice demonstrated a decreased percentage of Eomes^+^, PD-1^+^ Eomes^+^, and PD-1^+^ KLRG1^−^ LCMV-specific CD8^+^ T cells. CD8^+^ T cells from both short term ASP1570 treatment mice and ERK^SEM^ mice degranulated and produced more IFNγ upon LCMV peptide restimulation. An increase in T cell activation correlated with a decreased LCMV virus titer at Day 35 post infection. These data suggest that the selective enhancement of ERK or short-term inhibition of DGKζ during the chronic phase of LCMV CL13 infection reinvigorates exhausted T cells (**Figure 10**).

Our study revealed that the enhancement of DAG signaling during early T cell activation promotes SLEC differentiation and subsequent T cell death at the expense of survival and memory phenotype T cell formation during chronic LCMV infection. However, the selective activation of ERK pathway promoted the survival and increased the fraction of memory phenotype T cells, which correlated with decreased T cell exhaustion and improved viral control in the chronic phase of LCMV infection. Short term inhibition of DGK ζ provided benefit in the anti-virus response during acute infection and reinvigorated T cells during chronic infection. Thus, our study positions the enhancement of ERK signaling as a potential target for enhancing T cell responses undergoing chronic antigen stimulation. Furthermore, carefully timed manipulation of DAG-mediated signaling could also be beneficial in reversing T cell exhaustion.

## Data availability statement

The original contributions presented in the study are included in the article/**Supplementary Materials**. Further inquiries can be directed to the corresponding author.

## Ethics statement

The animal study was reviewed and approved by University of Pennsylvania.

## Author contributions

SH and OK performed experiments. SH, OK, HB, and TK designed experiments. SH and TK wrote the manuscript. OK, HB, TY, YO, MT, AK, and OI provided key reagents and edited the manuscript. All authors contributed to the article and approved the submitted version.

## Funding

This work was supported by funds from Astellas Pharma and the National Institutes of Health, R01HL111501 and R01HL146645 to TK and funds from The Ito Foundation and Society for Promotion of International Oto-Rhino-Laryngology (SPIO) to SH.

## Acknowledgments

We thank Mariko Okumura, Dr. Jennifer Wu, Dr. Jean-Christophe Beltra, Dr. Melanie Mumau, and Dr. Shin Ngiow for technical advice and support.

## Conflict of interest

TY, YO, MT, OI were employed by Astellas Pharma Inc.

The remaining authors declare that the research was conducted in the absence of any commercial or financial relationships that could be construed as a potential conflict of interest.

This work was sponsored by Astellas Pharma, who developed ASP1570.

## Publisher’s note

All claims expressed in this article are solely those of the authors and do not necessarily represent those of their affiliated organizations, or those of the publisher, the editors and the reviewers. Any product that may be evaluated in this article, or claim that may be made by its manufacturer, is not guaranteed or endorsed by the publisher.
