# Supplementary material for: Manipulation of diacylglycerol and ERK-mediated signaling differentially controls CD8+ T cell responses during chronic viral infection
Source: Front Immunol. 2022 Nov 24;13:1032113. doi: 10.3389/fimmu.2022.1032113 (PMC9951774; doi:10.3389/fimmu.2022.1032113)
Supplement: Supplementary file 1 [file DataSheet_1.pdf]

Target	Clone	Fluorochrome	Company
Bcl-2	BCL/10C4	PE	Biolegend
Bcl-2	10C4	PE-Cy7	eBioscience
Bim	H-5	FITC	Santa Cruz
CD107a (LAMP-1)	1D4B	PE	Biolegend
CD127	A7R34	BV 711	Biolegend
CD223 (Lag3)	C9B7W	BV711	BD Biosciences
CD244.2 (2B4)	eBio244F4	FITC	eBioscience
CD279 (PD-1)	29F.1A12	PE-Cy7	Biolengend
CD279 (PD-1)	29F.1A12	BV421	Biolengend
CD279 (PD-1)	29F.1A12	PE	Biolengend
CD366 (Tim3)	RMT3-23	CD605	Biolegend
CD4	RM4-5	APC-eF780	eBiocience
CD44	IM7	BV711	Biolegend
CD45R (B220)	RA3-6B2	APC-eF780	eBiocience
CD45.1	A20	PE-Cy7	Biolegend
CD62L	MEL-14	BV605	Biolegend
CD8a	53-6.7	PE-eF610	eBiocience
CD8a	53-6.7	BV421	Biolegend
CD8a	53-6.7	BV650	Biolegend
CD8a	SK1	BV711	Biolegend
CD90.2 (Thy1.2)	53-2.1	APC	eBioscience
Eomes	Dan11mg	PE-eF610	Biolegend
IFNγ	4S.B3	PE-eF610	eBioscience
KLRG1	2F1	FITC	Southern Biotech
pERK	6B8B69	PE	Biolegend
pS6	A17029B	PECy7	Biolegend
pS6	D57.2.2E	PECy7	Cell signaling
Tigit	1G9	BV421	BD Biosciences
Dead cell	Live/Dead Near-IR		ThermoFisher
LCMV GP33 tetramer	KAVYNFATC	PE	MBL international
LCMV GP276 tetramer	SGVENPGGYCL	APC	MBL international
CD3e	145-2C11	-	BD Biosciences

Supplementary Table 1. Flow cytometry antibodies. List of fluorophore-conjugated antibodies used for surface staining for flow cytometry and cell sorting.

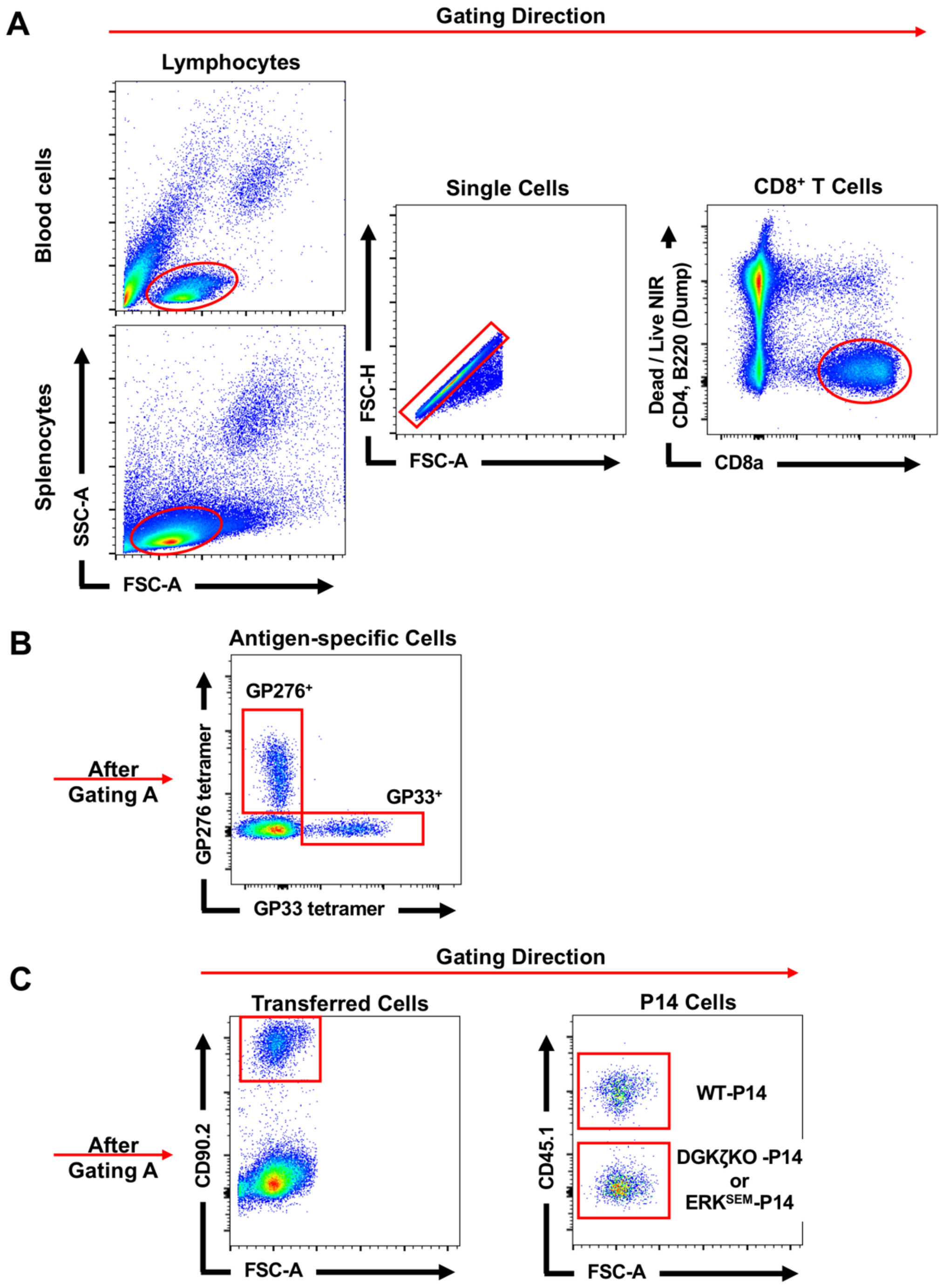

Supplementary Figure 1. Gating strategy employed for flow cytometry. (A) Blood cells or splenocytes were gated by FSC and SSC, followed by live single cells, and CD8^{+} T cell gating. **(B)** Gated CD8^{+} T cells were gated on GP33-tetramer or GP276-tetramer-positive cells. **(C)** In adoptive transfer studies, single CD8^{+} T cells were gated on Thy1.2 followed by either CD45.1^{+} or CD45.2^{+} cell gating.

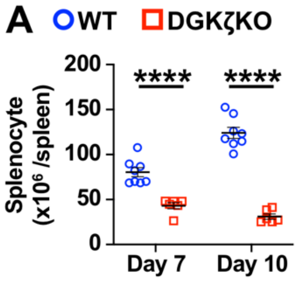

Supplementary Figure 2. Splenocytes from DGKζ KO mice are decreased after LCMV CL13 infection. **(A)** The absolute number of splenocytes were counted in WT and DGKζ KO mice at Days 7 and 10 post LCMV CL13 infection. ****p<0.0001 by Student t-test.

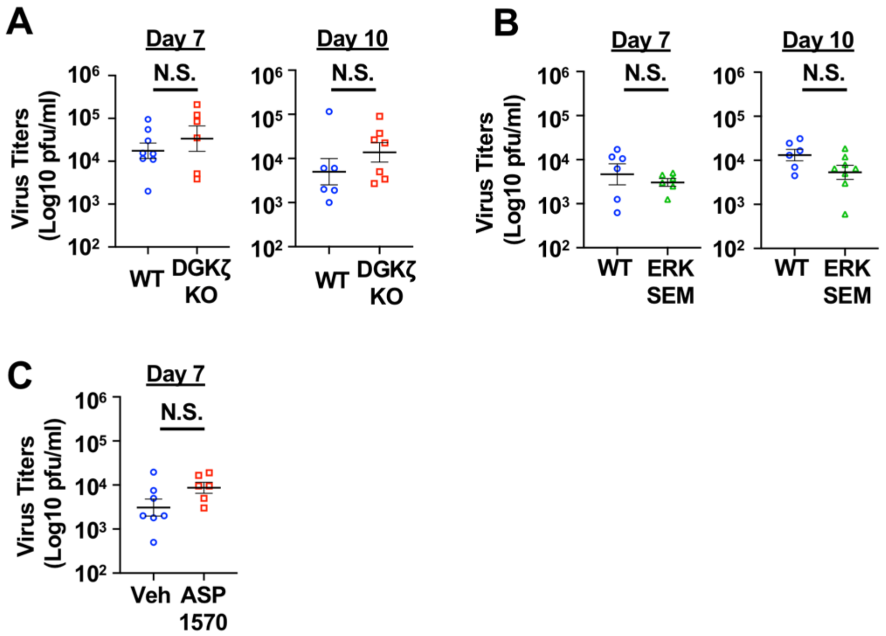

Supplementary Figure 3. Serum virus titers. (A) Virus titers of WT and DGKζ KO mice on Days 7 and 10 post LCMV CL13 infection. **(B)** Virus titers of WT and ERK^{SEM} mice on Day 7 and 10 post LCMV CL13 infection. **(C)** Virus titers of vehicle and ASP1570-treated mice on Day 7 post LCMV CL13 infection. N.S. = not significant by Student t-test.

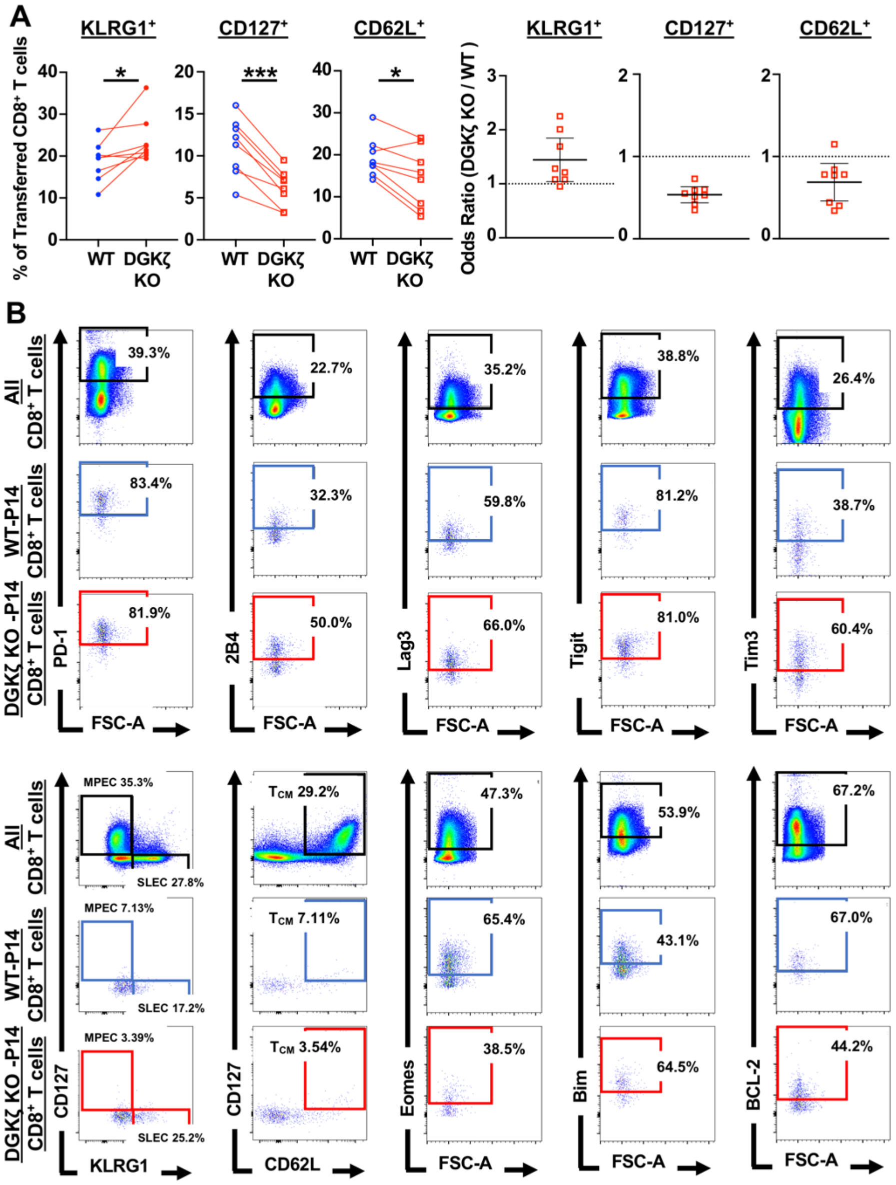

Supplementary Figure 4. Expression of cell surface markers on DGKζ KO LCMV-specific T cells. (A) CD8$^+$ T cells from WT-P14 (CD45.1$^+$) and DGKζ KO-P14 (CD45.2$^+$) mice were mixed at a 1:1 ratio and adoptively transferred into Thy1.1$^+$ WT host mice 1 day before infection with LCMV CL13. The fraction and odds ratio of WT and DGKζ KO T cells expressing KLRG1, CD127, or CD62L were quantified at Day 10 post LCMV CL13 infection. Data from N=6 mice/group pooled from 2 independent experiments are shown. N.S. = not significant, *p<0.05, **P<0.01, ****p<0.0001 by paired Student t-test. **(B)** Representative flow cytometry plots of all CD8$^+$ T cells, WT P14 CD8$^+$ T cells, and DGKζ KO P14 CD8$^+$ T cells expressing PD-1, 2B4, LAG3, TIGIT, TIM3, KLRG1, CD127, CD62L, Eomes, Bim, BCL-2 is shown.

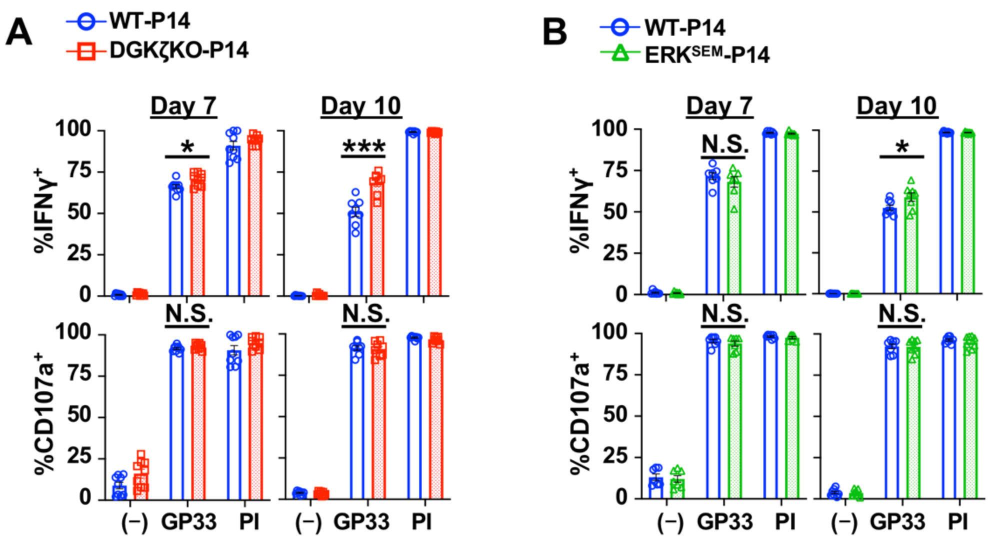

Supplementary Figure 5. Restimulation of P14 T cells. (A) CD8$^+$ T cells from WT-P14 (CD45.1$^+$) and DGKζ KO-P14 (CD45.2$^+$) mice were mixed at a 1:1 ratio and adoptively transferred into Thy1.1$^+$ WT host mice 1 day before infection with LCMV CL13. On Days 7 and 10 post infection, splenocytes were restimulated with GP33 peptide or PMA/ionomycin (PI). The fraction of CD8$^+$ T cells expressing IFNγ or CD107a was quantified. **(B)** CD8$^+$ T cells from WT-P14 (CD45.1$^+$) and ERK^{SEM} P14 (CD45.2$^+$) mice were mixed at a 1:1 ratio and adoptively transferred into Thy1.1$^+$ WT host mice 1 day before infection with LCMV CL13. On Days 7 and 10 post infection, splenocytes were restimulated with GP33 peptide or PMA/ionomycin (PI). The fraction of CD8$^+$ T cells expressing IFNγ or CD107a was quantified. N.S. = not significant, *p<0.05, ***p<0.001 by paired Student t-test.

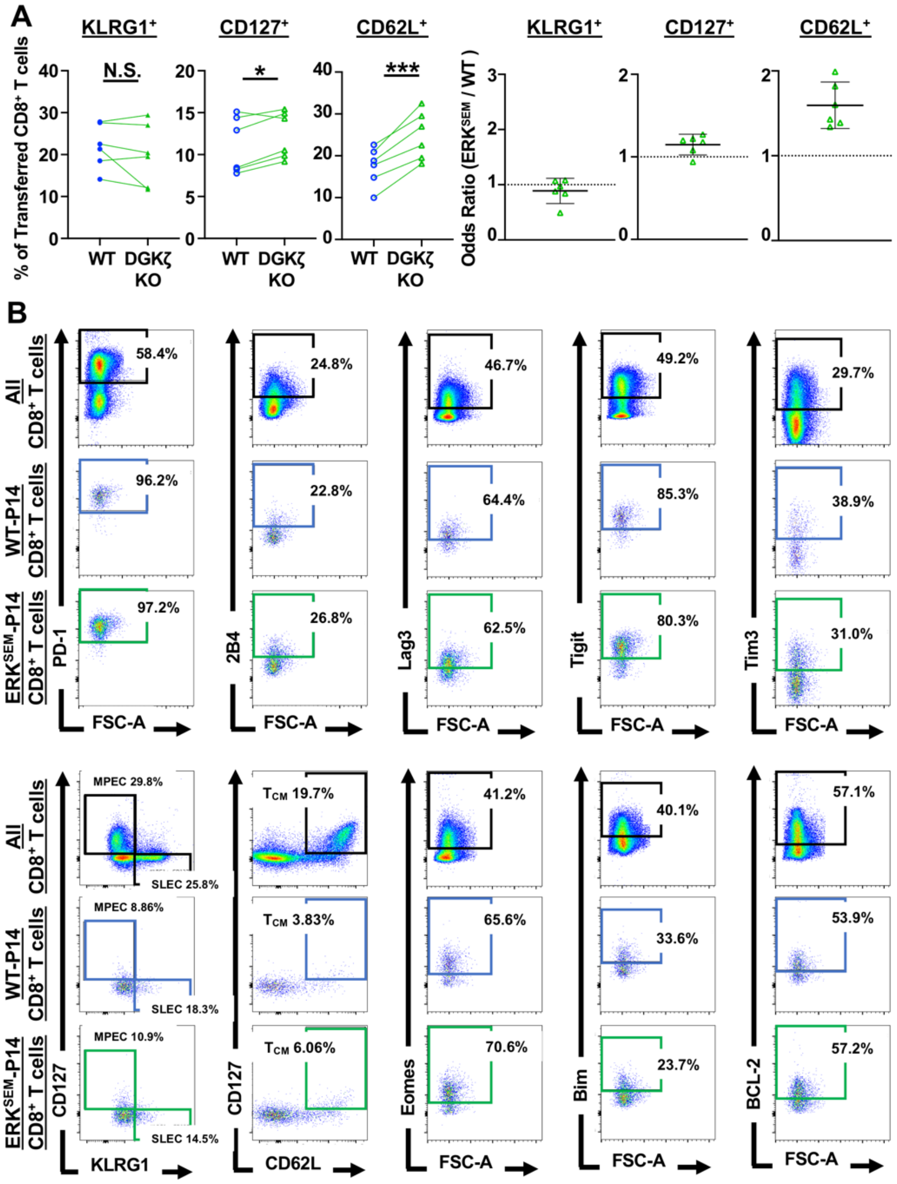

Supplementary Figure 6. Expression of cell surface markers on ERK^{SEM} LCMV-specific T cells. (A) CD8$^+$ T cells from WT-P14 (CD45.1$^+$) and ERK^{SEM}-P14 (CD45.2$^+$) mice were mixed at a 1:1 ratio and adoptively transferred into Thy1.1$^+$ WT host mice 1 day before infection with LCMV CL13. The fraction and odds ratio of WT and ERK^{SEM} T cells expressing KLRG1, CD127, or CD62L were quantified at Day 10 post LCMV CL13 infection. Data from N=6 mice/group pooled from 2 independent experiments are shown. N.S. = not significant, *p<0.05, **P<0.01, ****p<0.0001 by paired Student t-test. (B) Representative flow cytometry plots of all CD8$^+$ T cells, WT P14 CD8$^+$ T cells, and ERK^{SEM} P14 CD8$^+$ T cells expressing PD-1, 2B4, LAG3, TIGIT, TIM3, KLRG1, CD127, CD62L, Eomes, Bim, BCL-2 is shown.

A

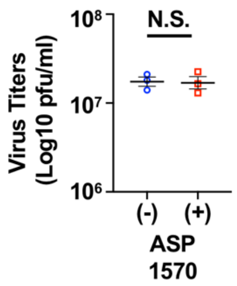

Supplementary Figure 7. The effect of ASP1570 on LCMV CL13 plaque assay. An LCMV CL13 plaque assay was performed in the presence or absence of ASP1570 (1 µM). N.S. = not significant by Student t-test.

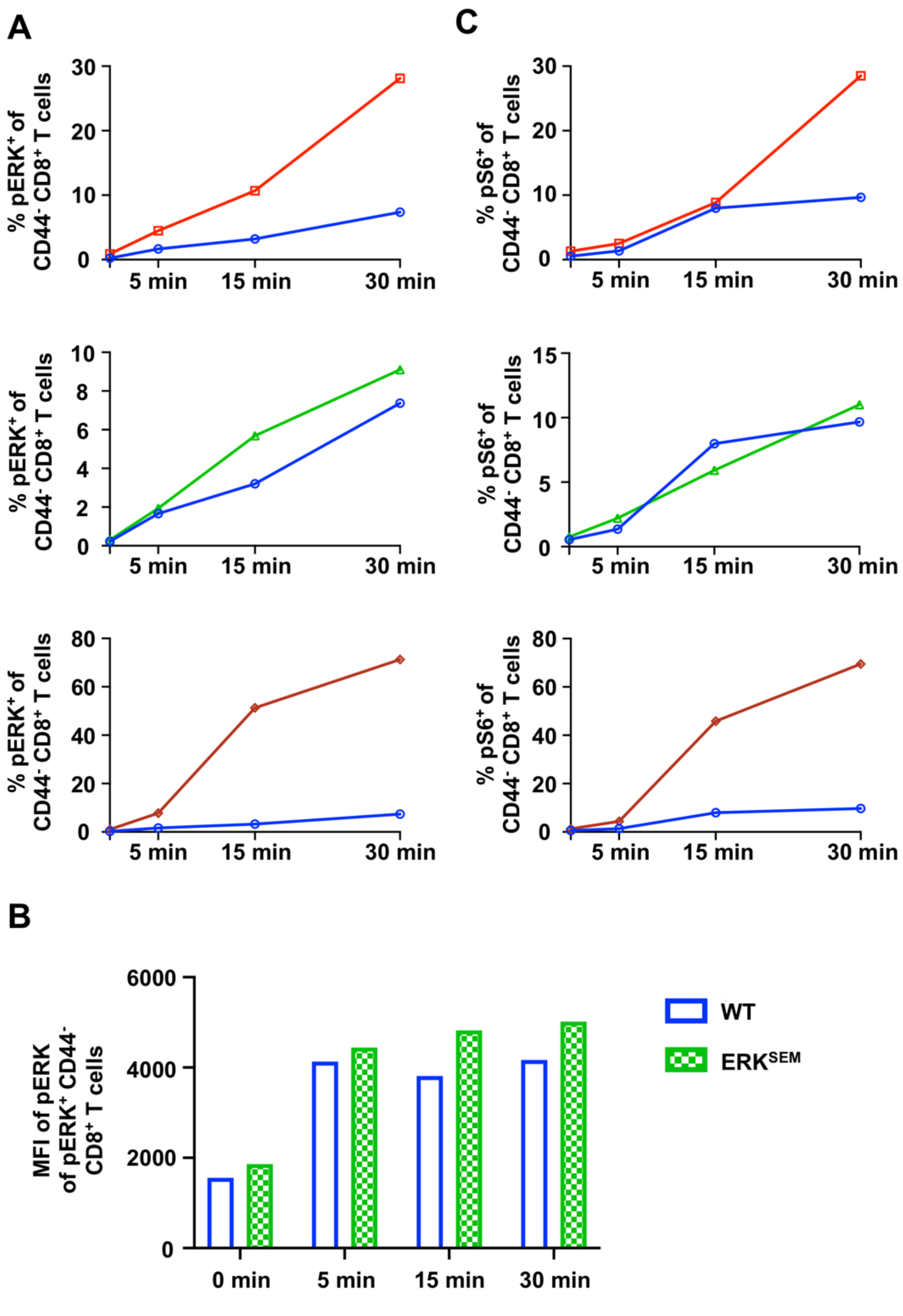

Supplementary Figure 8. Phosphorylation of ERK and S6 in TCR-stimulated DGKζ KO, ERK^{SEM}, and ASP1570-treated CD8+ T cells. Splenocytes were stimulated with anti-CD3 antibody for the indicated times and **(A)** the fraction of CD44^{lo} CD8+ T cells displaying pERK, **(B)** the MFI of pERK in pERK+ cells of WT and ERK^{SEM}, and **(C)** the fraction of CD44^{lo} CD8+ T cells displaying pS6 was quantified by flow cytometry.
